# Inhibition of adenosine A1 receptors abolished the nutritional ketosis-evoked delay in the onset of isoflurane-induced anesthesia in Wistar Albino Glaxo Rijswijk rats

**DOI:** 10.1186/s12871-020-0943-z

**Published:** 2020-01-30

**Authors:** Zsolt Kovács, Brigitta Brunner, Dominic P. D’Agostino, Csilla Ari

**Affiliations:** 1 Savaria Department of Biology, ELTE Eötvös Loránd University, Savaria University Centre, Szombathely, Hungary; 2 Institute of Biology, University of Pécs, Pécs, Hungary; 3 Department of Molecular Pharmacology and Physiology, Laboratory of Metabolic Medicine, Morsani College of Medicine, University of South Florida, Tampa, FL USA; 4 Institute for Human and Machine Cognition, Ocala, FL USA; 5 Department of Psychology, Hyperbaric Neuroscience Research Laboratory, University of South Florida, 4202 E. Fowler Ave, PCD 3127, Tampa, FL 33620 USA

**Keywords:** Isoflurane, Anesthesia, Exogenous ketone supplements, Ketosis, Adenosine receptors

## Abstract

**Background:**

It has been demonstrated that administration of exogenous ketone supplement ketone salt (KS) and ketone ester (KE) increased blood ketone level and delayed the onset of isoflurane-induced anesthesia in different rodent models, such as Wistar Albino Glaxo Rijswijk (WAG/Rij) rats. The modulatory effect of adenosinergic system may have a role in the ketone supplementation-evoked effects on isoflurane-generated anesthesia. Thus, we investigated whether adenosine receptor antagonists can modulate the effect of exogenous ketone supplements on the onset of akinesia induced by isoflurane.

**Methods:**

To investigate the effect of exogenous ketone supplements on anesthetic induction we used ketone supplement KE, KS, KEKS (1:1 mix of KE and KS), KSMCT and KEMCT (1:1 mix of KS and KE with medium chain triglyceride/MCT oil, respectively) in WAG/Rij rats. Animals were fed with standard diet (SD), which was supplemented by oral gavage of different ketone supplements (2.5 g/kg/day) for 1 week. After 7 days, isoflurane (3%) was administered for 5 min and the time until onset of isoflurane-induced anesthesia (time until immobility; light phase of anesthesia: loss of consciousness without movement) was measured. Changes in levels of blood β-hydroxybutyrate (βHB), blood glucose and body weight of animals were also recorded. To investigate the putative effects of adenosine receptors on ketone supplements-evoked influence on isoflurane-induced anesthesia we used a specific adenosine A1 receptor antagonist DPCPX (intraperitoneally/i.p. 0.2 mg/kg) and a selective adenosine A2A receptor antagonist SCH 58261 (i.p. 0.5 mg/kg) alone as well as in combination with KEKS.

**Results:**

Significant increases were demonstrated in both blood βHB levels and the number of seconds required before isoflurane-induced anesthesia (immobility) after the final treatment by all exogenous ketone supplements. Moreover, this effect of exogenous ketone supplements positively correlated with blood βHB levels. It was also demonstrated that DPCPX completely abolished the effect of KEKS on isoflurane-induced anesthesia (time until immobility), but not SCH 58261.

**Conclusions:**

These findings strengthen our previous suggestion that exogenous ketone supplements may modulate the isoflurane-induced onset of anesthesia (immobility), likely through A1Rs.

## Background

It has been demonstrated that exogenous ketone (ketogenic) supplements, such as ketone ester (KE), not only increase the level of ketone bodies (e.g., β-hydroxybutyrate/βHB) [1–5], but also maintain blood levels of ketone bodies in both animals and humans [2, 3, 6]. Ketone bodies, such as βHB, enter into the brain through blood-brain barrier and provide fuel to brain cells [7, 8] improving cell energy metabolism (e.g., enhance mitochondrial ATP synthesis) [9]. Moreover, ketone supplement-induced ketosis can suppress neuronal excitability [7, 10, 11], modulate functions of ion channels and neurotransmitter systems (e.g., increase GABA and adenosine levels) [7, 12–14] and influence inflammatory processes (e.g., decrease the concentration and expression of proinflammatory cytokines) [15]. It was suggested that these effects of ketosis may have therapeutic potential in the treatment of several central nervous system (CNS) diseases, such as Alzheimer’s disease, Parkinson’s disease, epilepsy and psychiatric disorders (e.g., anxiety, schizophrenia and depression) [1, 3, 8, 16]. It was also demonstrated that exogenous ketone supplements, such as KE and ketone salt (KS) are relatively well-tolerated without (or with minimal) adverse effects [1, 2, 6, 16, 17]. However, exact mechanism(s) of action of exogenous ketone supplement-generated ketosis on CNS diseases and other pathophysiological and physiological processes are largely unknown.

It was suggested that ketosis may modulate sleep and sleep-like effects [18–22]. Indeed, it has been demonstrated recently that nutritional ketosis (evoked by exogenous ketone supplements, such as KE) delayed the onset of inhalational anesthetics isoflurane (1-chloro-2,2,2-trifluoroethyl difluoromethyl ether)- induced anesthesia (immobility) [23] (light phase of anesthesia: loss of consciousness without movement, which was defined as ‘immobility’) [24]. Nevertheless, mechanism of action of ketosis-induced changes in isoflurane-evoked anesthesia remains unknown. It was suggested that changes, for example, in functioning of different ion channels (e.g., K_ATP_ channels), neurotransmitter systems (e.g., GABAergic and adenosinergic system) and mitochondria (e.g., mitochondrial respiration) may have a role in ketone supplement-evoked effects on isoflurane-generated anesthesia [19, 23, 25–27]. However, it has also been demonstrated that ketosis (evoked by exogenous ketone supplements) [1, 2, 4, 5] may increase adenosine level in the brain [14] and adenosine may have a role not only in the sleep [28], but also the generation of sleep-like effects [29, 30]. Therefore, in this study, we examined the effect of ketone supplement KE, KS and their mix (KEKS), as well as mix of KS and KE with medium chain triglyceride (MCT) oil (KSMCT and KEMCT, respectively) on isoflurane-induced onset of anesthesia (latency to immobility). Animals (Wistar Albino Glaxo Rijswijk/WAG/Rij rats) were fed with standard diet (SD) and were gavaged with different ketogenic supplements for 1 week (2.5 g/kg/day). After the last supplement gavage we recorded the time until onset of immobility (under 3% isoflurane). In the second part of the study, the potential role of adenosine receptors in the nutritional ketosis-evoked effects on isoflurane-induced onset of anesthesia (immobility) was investigated. We used a specific adenosine A1 receptor (A1R) antagonist DPCPX (1,3-dipropyl-8-cyclopentylxanthine) (intraperitoneally/i.p. 0.2 mg/kg) and a selective adenosine A2A receptor (A2AR) antagonist SCH 58261 (7-(2-phenylethyl)-5-amino-2-(2-furyl)-pyrazolo-[4,3-e]-1,2,4-triazolo[1,5-c]pyrimidine) (i.p. 0.5 mg/kg) alone as well as in combination with KEKS (2.5 g/kg/day, gavage).

This study is the continuation of our previous study on genetically absence epileptic WAG/Rij rat strain (a well-investigated model of human absence epilepsy) [31], in which it was demonstrated that exogenous ketone supplements (such as KE) delayed the onset of isoflurane-induced anesthesia (increased the time required before immobility) [23]. These effects may be clinically relevant because administration of exogenous ketone supplements-induced ketosis are more and more widely used as a metabolic therapy in the treatment of different CNS diseases, such as epilepsy or other seizure disorders [2, 8, 32–35]. Consequently, in order to implement a safe and successful anesthesia, potential effects of ketosis on the latency to anesthesia might need to be considered when epileptic patients are undergoing anesthetic procedures. For this reason, this study was performed on WAG/Rij rats, to better understand the ketone supplement-evoked effects on isoflurane-generated onset of anesthesia and its mechanism of action under epileptic condition.

In this study we hypothesized that adenosine receptor inhibition may modulate the exogenous ketone supplement-evoked delay in the latency to onset of immobility.

## Methods

### Animals

Animal treatments were carried out according to the Hungarian Act of Animal Care and Experimentation (1998, XXVIII, section 243), European Communities Council Directive 24 November 1986 (86/609/EEC) and EU Directive 2010/63/EU to use and treat animals in experimental laboratories. The experimental design was approved by the Animal Care and Experimentation Committee of the Eötvös Loránd University (Savaria University Centre) and National Scientific Ethical Committee on Animal Experimentation (Hungary) under license number VA/ÉBNTF02/85–8/2016.

Male WAG/Rij rats (*n* = 80; 6 months old, 315–332 g; breeding colony of WAG/Rij rats at Eötvös Loránd University, Savaria University Centre, Szombathely, Hungary) were kept in groups of 3–4 under standard laboratory conditions (12:12 h light-dark cycle, light was on from 08.00 AM to 08.00 PM; free access to food and water; air-conditioned room at 22 ± 2 °C). Rats were fed with standard rodent chow diet (SD), and received oral (intragastric) gavage of either water (control) or different ketone supplements (KE, KS, KSMCT, KEKS or KEMCT). The animals were euthanized after the last treatment and data collection by using isoflurane. All efforts were made to minimize pain and suffering and to reduce the number of animals used.

### Treatment groups and detection of immobility

Both KE (1,3-butanediol – acetoacetate diester) and KS (Na^+^/K^+^ − βHB mineral salt) were developed by D’Agostino et al. [2] (University of South Florida/USF, United States) in collaboration with Savind, Inc. (Urbana, IL, United States). Ketone salt was mixed into a 50% solution (375 mg/g pure βHB and 125 mg/g of Na^+^/K^+^ in a 1:1 ratio). Medium chain triglyceride (MCT) oil (pharmaceutical grade; approximately 60% caprylic triglyceride and 40% capric triglyceride) was purchased from Now Foods (Bloomingdale, IL, United States).

We demonstrated previously the tolerability and effectiveness of exogenous ketone supplements KE, KS, KSMCT (mix of KS and MCT oil in a 1:1 ratio), KEKS (mix of KE and KS in a 1:1 ratio) and KEMCT (mix of KE and MCT oil in a 1:1 ratio) given by intragastric gavage (ad libitum access to normal rat chow + 2.5 g/kg body weight supplements by gavage once/day in WAG/Rij rats) [1, 4, 32, 36]. Mix of ketone supplements (KSMCT, KEKS, and KEMCT) was carried out at the Eötvös Loránd University (Savaria University Centre, HUNGARY). These types and dose of ketone supplements introduced by oral gavage once per day for 7 days effectively induced and maintained ketosis in our previous studies [1, 32, 36] without causing side effects. Therefore, in the first phase of this study, 2.5 g/kg/day dosage of ketone supplements (KE, KS, KSMCT, KEKS and KEMCT) was administered daily by gavage for 7 days. In the second phase of the study, to investigate the putative adenosinergic mechanism of action of ketone supplements on isoflurane-evoked anesthesia (latency to immobility), we also used a specific A1R antagonist DPCPX and a selective A2AR antagonist SCH 58261, which drugs were dissolved in 10% dimethyl sulfoxide (DMSO). All drugs were purchased from Sigma-Aldrich Inc. (Hungary, Budapest). In order to minimize the putative adverse effects of drugs and to induce antagonism of A1Rs and A2ARs without changes in absence epileptic activity we used previously tested and effective i.p. dose of DPCPX and SCH 58261 (0.2 mg/kg DPCPX and 0.5 mg/kg SCH 58261) alone as well as in combination with KEKS (2.5 g/kg/day, gavage) [32, 37, 38]. Moreover, it was demonstrated previously that 10% of DMSO alone has no effect on absence epileptic activity (spike-wave discharges, SWDs) in WAG/Rij rats [39] and on sleep architecture in rats [40].

Oral gavage is a relatively stressful administration method, which may affect the sensitivity of animals to anesthetics [41]. Thus, to familiarize the animals to the methods, the 7 days gavage treatment was preceded by i.p. injection of 0.5 ml saline/100 g body weight and (30 min later) by water gavage for 5 days (adaptation period). Following adaptation period, rats were randomly assigned into 10 groups with 8 animals in each group. All of the rats were injected i.p. by 0.5 ml saline/100 g body weight/every day 30 min before gavage. After the i.p. injection, water (2.5 g/kg body weight/day, group 1; SD, control group) or exogenous ketone supplements (KE, KS, KSMCT, KEKS or KEMCT: 2.5 g/kg body weight/day; group 2–6, respectively) were administered by gavage for 7 days. One hour after the 7th treatments, anesthesia was induced in an air tight anesthesia chamber with isoflurane (3% isoflurane gas mixed with air for 5 min). Time from chamber closure until immobility (loss of consciousness without movement) was measured and analyzed by a blinded observer similar to previously [23]. To test the putative effect of DPCPX and SCH 58261 alone on isoflurane-induced anesthesia (latency to immobility), animals were i.p. injected and gavaged similar to group 1, but on the 7th treatment (water gavage) day, i.p. injections contained 0.2 mg/kg DPCPX (group 7) or 0.5 mg/kg SCH 58261 (group 8) in 0.5 ml 10% DMSO solution/100 g body weight. Based on results on group 2–6, the most effective ketone supplement (KEKS) was chosen for investigation of the putative mechanism of action. Therefore, animals (group 9 and group 10) were i.p. injected by saline and gavaged for 7 days by KEKS similar to described above, but on the 7th treatment (KEKS gavage) day, the i.p. injections contained 0.2 mg/kg DPCPX (group 9) or 0.5 mg/kg SCH 58261 (group 10) in 0.5 ml 10% DMSO solution/100 g body weight. After administration of i.p. DPCPX (group 7) and SCH 58261 (group 8) alone as well as combined administration of KEKS with i.p. DPCPX (group 9) or SCH 58261 (group 10), anesthesia was induced and immobility was measured similar to group 1–6 on the 7th treatment days.

Each rat was used only in one of the treatment groups and was euthanized with isoflurane after the 7th treatment and data collection.

### Measurement of blood βHB and glucose levels as well as body weight

Blood was taken from the tail vein of rats. βHB levels were measured by a commercially available glucose and ketone monitoring system (Precision Xtra™, Abbott Laboratories, Abbott Park, IL, USA) [1, 23, 32]. Total blood ketone levels (D-βHB + L-βHB + acetoacetate + acetone) would be higher than we measured because this instrument only measures blood levels of D-βHB. Baseline βHB and glucose levels were measured on the last (5th) day of the adaptation period (group 1–6). Levels of βHB and glucose were measured again after the last (7th) day of water (group 1, control) and ketone supplementation (gavage; group 2–6) on awake animals, approximately 10 min after the detection of isoflurane-induced anesthesia (immobility) [23].

Body weight of rats were measured before (on last/5th day of the adaptation period) and after (on last/7th day of gavage) the treatments (group 1–6).

### Statistics

All data were presented as the mean ± standard error of the mean (S.E.M.). We compared the latency of isoflurane-induced anesthesia (immobility) in control group (SD; group 1; gavaged by water for 7 days) and treated groups (gavaged by different exogenous ketone supplements for 7 days: group 2–6; i.p. injected by DPCPX or SCH 58261 alone on 7th treatment days: group 7, and group 8; administration of KEKS in combination with i.p. DPCPX or SCH 58261 on 7th treatment days: group 9, and group 10). Moreover, baseline (last/5th day of the adaptation period; group 1–6), control (SD; group 1; 7th day) and ketone supplements-induced (group 2–6; 7th day) blood glucose and βHB levels as well as body weight (before treatment and after treatment: group 1–6) were also compared. Data analysis was performed using GraphPad Prism version 6.0a using a two-way ANOVA with Tukey’s multiple comparisons test. Pearson correlation was calculated for blood βHB and anesthesia latency as individual data points and as group means [23]. Results were considered significant when *p* < 0.05.

## Results

### Effects of exogenous ketone supplements on blood βHB and glucose levels and body weight

**Fig. 1:**
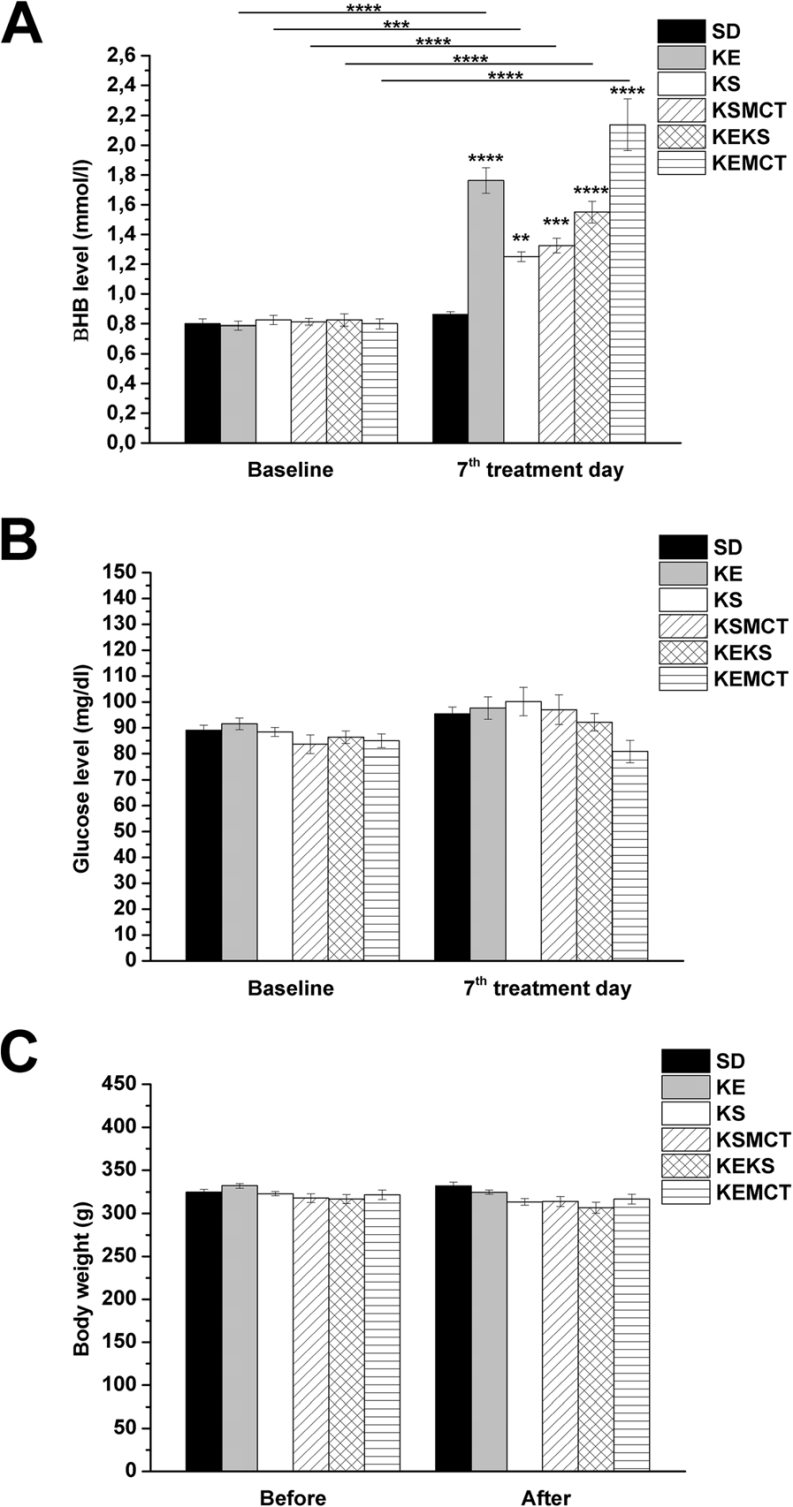
Ketone supplements-evoked changes in blood βHB and glucose levels as well as body weight. Blood βHB levels were significantly elevated in all groups (group 2–6) gavaged by ketone supplements (KE, KS, KSMCT, KEKS and KEMCT) for 7 days, compared to both control (standard diet/SD, gavaged with water for 7 days) and to their baseline (**a**). After different treatments, glucose level (**b**) and body weight (**c**) of animals did not change compared to both control (SD) and to their baseline. Abbreviations: After, after the treatments; Before, before the treatments; KE, ketone ester; KEKS, mix of KE and KS in a 1:1 ratio; KEMCT, mix of KE and medium chain triglyceride (MCT) oil in a 1:1 ratio; KS, ketone salt; KSMCT, mix of KS and MCT oil in a 1:1 ratio; SD, standard diet/control; **p < 0.01; ***p < 0.001; ****p < 0.0001

A significant increase in blood βHB levels was demonstrated after the final (7th) treatment by all exogenous ketone supplements (KE, KS, KSMCT, KEKS and KEMCT; group 2–6), compared to both control (SD; *p* < 0.01 for KS; *p* < 0.001 for KSMCT; *p* < 0.0001 for KE, KEKS and KEMCT) and baseline (p < 0.001 for KS; p < 0.0001 for KE, KSMCT, KEKS and KEMCT) levels (Fig. 1a; Table 1).

**Table 1:** Effect of ketone supplements on blood βHB and glucose levels on the 7th day of gavage

	Blood βHB level			
Treatments (2.5 g/kg/day; Fig. 1a)	Baseline	7th treatment day		
	mmol/l (mean ± S.E.M.)	mmol/l (mean ± S.E.M.)	Compared to baseline (significance level/q-value)	Compared to control (significance level/q-value)
SD (control; group 1)	0.80 ± 0.037	0.86 ± 0.018	−/0.945	–
KE (group 2)	0.79 ± 0.029	1.76 ± 0.087	****/14.550	****/13.610
KS (group 3)	0.83 ± 0.031	1.25 ± 0.033	***/6.805	**/5.860
KSMCT (group 4)	0.81 ± 0.023	1.33 ± 0.049	****/7.939	***/6.994
KEKS (group 5)	0.83 ± 0.041	1.55 ± 0.073	****/11.340	****/10.400
KEMCT (group 6)	0.80 ± 0.033	2.14 ± 0.172	****/20.230	****/19.280
	Blood glucose level			
Treatments (2.5 g/kg/day; Fig. 1b)	Baseline	7th treatment day		
	mg/dl (mean ± S.E.M.)	mg/dl (mean ± S.E.M.)	Compared to baseline (significance level/q-value)	Compared to control (significance level/q-value)
SD (control; group 1)	89.00 ± 1.937	95.38 ± 2.672	−/1.764	–
KE (group 2)	91.50 ± 2.284	97.63 ± 4.342	−/2.386	−/0.623
KS (group 3)	88.38 ± 1.772	100.13 ± 5.482	−/3.078	−/1.314
KSMCT (group 4)	83.63 ± 3.600	97.00 ± 5.745	−/2.213	−/0.449
KEKS (group 5)	86.38 ± 2.398	92.13 ± 3.388	−/0.865	−/0.899
KEMCT (group 6)	85.00 ± 2.659	80.88 ± 4.286	−/2.248	−/4.011

Abbreviations: KE, ketone ester; KEKS, mix of KE and KS in a 1:1 ratio; KEMCT, mix of KE and medium chain triglyceride (MCT) oil in a 1:1 ratio; KS, ketone salt; KSMCT, mix of KS and MCT oil in a 1:1 ratio; SD, standard diet/control; **p < 0.01; ***p < 0.001; ****p < 0.0001

After the 7th treatment day, changes in glucose levels and body weight of animals were not detected (Fig. 1b and c; Table 1 and Table 2).

**Table 2:** Effect of ketone supplements on body weight

	Body weight		
Treatments (2.5 g/kg/day; Fig. 1)	Before the treatments	After the treatments	
	Gram (mean ± S.E.M.)	Gram (mean ± S.E.M.)	Compared to ‘Before the treatments’ (significance level/q-value)
SD (control, group 1)	325.0 ± 3.134	331.8 ± 4.427	−/1.464
KE (group 2)	331.9 ± 2.496	324.9 ± 2.608	−/0.027
KS (group 3)	323.1 ± 2.394	313.5 ± 3.942	−/2.495
KSMCT (group 4)	318.0 ± 5.119	314.0 ± 5.719	−/2.386
KEKS (group 5)	316.9 ± 5.377	306.6 ± 6.434	−/3.986
KEMCT (group 6)	321.8 ± 5.502	316.8 ± 5.640	−/1.790

Abbreviations: KE, ketone ester; KEKS, mix of KE and KS in a 1:1 ratio; KEMCT, mix of KE and medium chain triglyceride (MCT) oil in a 1:1 ratio; KS, ketone salt; KSMCT, mix of KS and MCT oil in a 1:1 ratio; SD, standard diet/control

### Effect of exogenous ketone supplements on isoflurane-induced anesthesia: delay in the latency to onset of immobility

**Fig. 2:**
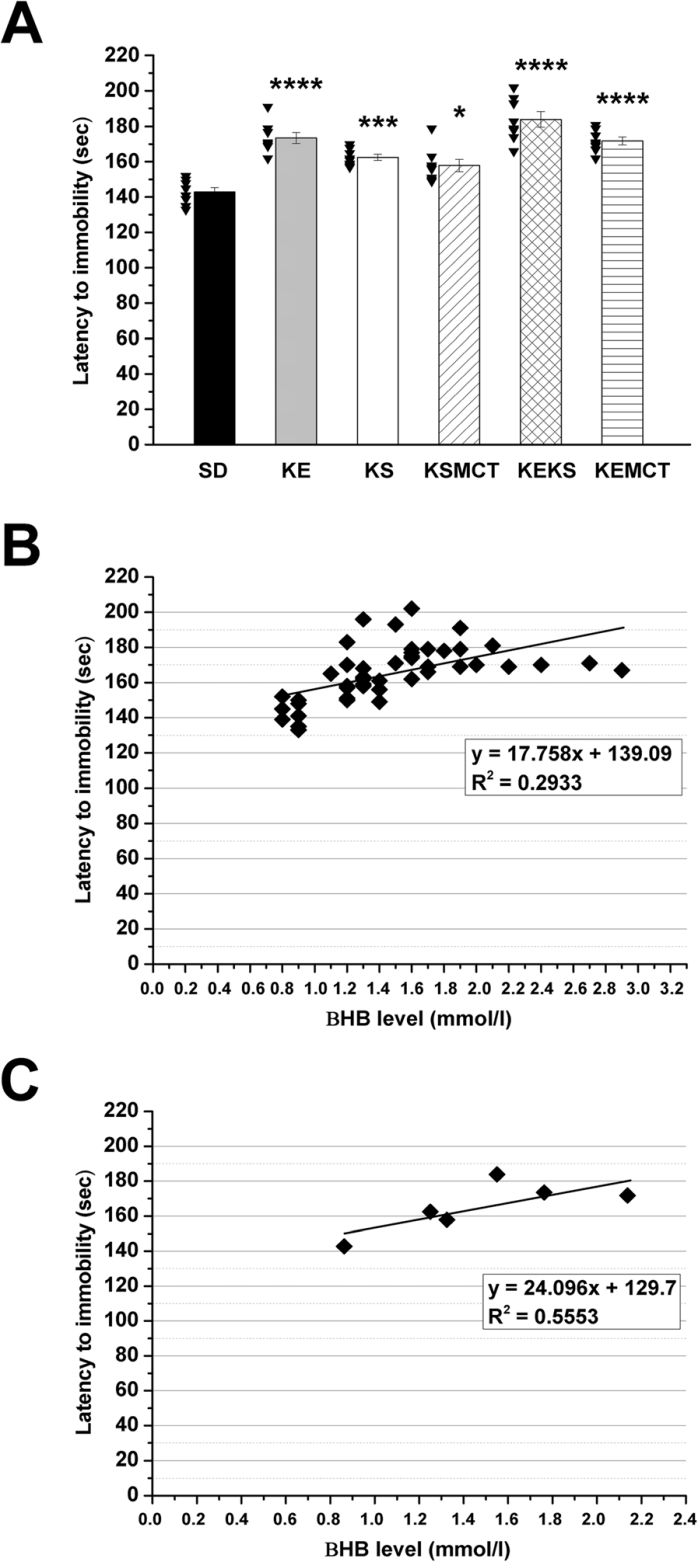
Effect of ketone supplements on isoflurane-induced anesthesia (latency to immobility). Gavage by exogenous ketone supplements (KE, KS, KSMCT, KEKS and KEMCT; group 2–6) significantly increased the latency to anesthetic induction (the time until immobility), compared to control (SD) on the 7th day of gavage (**a**; the raw data was plotted as filled black triangles to the left of the columns). There was a positive correlation between latency to immobility and blood βHB levels when all data point (**b**; R^2^ = 0.2933) or the group means (**c**; R^2^ = 0.5553) were considered. Abbreviations: KE, ketone ester; KEKS, mix of KE and KS in a 1:1 ratio; KEMCT, mix of KE and medium chain triglyceride (MCT) oil in a 1:1 ratio; KS, ketone salt; KSMCT, mix of KS and MCT oil in a 1:1 ratio; SD, standard diet/control; *p < 0.05; ***p < 0.001; ****p < 0.0001

Treatments by all exogenous ketone supplements (KE, KS, KSMCT, KEKS and KEMCT; group 2–6) caused a significant increase in the number of seconds required before anesthetic induction (the time until immobility), compared to control (SD; *p* < 0.05 for KSMCT; *p* < 0.001 for KS; *p* < 0.0001 for KE, KEKS and KEMCT) (Fig. 2a; Table 3) on the 7th day of treatment.

**Table 3:** Effect of ketone supplements, DPCPX, and SCH 58261 alone as well as ketone supplement KEKS in combination with DPCPX or SCH 58261 on latency to immobility on the 7th day of gavage

	Latency to immobility	
Treatments (2.5 g/kg/day; Figs. 2a and 3)	Sec (mean ± S.E.M.)	Compared to control (significance level/q-value)
SD (control, group 1)	142.88 ± 2.474	–
KE (group 2)	173.50 ± 3.105	****/10.220
KS (group 3)	162.50 ± 1.679	***/6.548
KSMCT (group 4)	157.88 ± 3.446	*/5.005
KEKS (group 5)	183.88 ± 4.299	****/13.680
KEMCT (group 6)	171.75 ± 2.226	****/9.634
DPCPX (group 7)	146.25 ± 4.443	−/0.765
SCH 58261 (group 8)	141.63 ± 4.935	−/0.283
KEKS + DPCPX (group 9)	138.13 ± 4.202	−/1.177
KEKS + SCH 58261 (group 10)	187.13 ± 5.531	****/12.290

Abbreviations: DPCPX (DP), 1,3-dipropyl-8-cyclopentylxanthine (a specific adenosine A1 receptor/A1R antagonist); KE, ketone ester; KEKS, mix of KE and KS in a 1:1 ratio; KEMCT, mix of KE and medium chain triglyceride (MCT) oil in a 1:1 ratio; KS, ketone salt; KSMCT, mix of KS and MCT oil in a 1:1 ratio; SCH (SC), SCH 58261, 7-(2-phenylethyl)-5-amino-2-(2-furyl)-pyrazolo-[4,3-e]-1,2,4-triazolo[1,5-c] pyrimidine (a selective adenosine A2A receptor/A2AR antagonist); SD, standard diet/control; *p < 0.05; ***p < 0.001; ****p < 0.0001

Exogenous ketone supplement-induced delay in isoflurane-generated anesthesia (increase in latency to immobility) positively correlated with blood βHB levels when individual data points (R^2^ = 0.2933) or the group means were considered (R^2^ = 0.5553) (Fig. 2b and c, respectively).

### Effect of A1R and A2AR inhibition on KEKS-evoked increase in latency to immobility

**Fig. 3:**
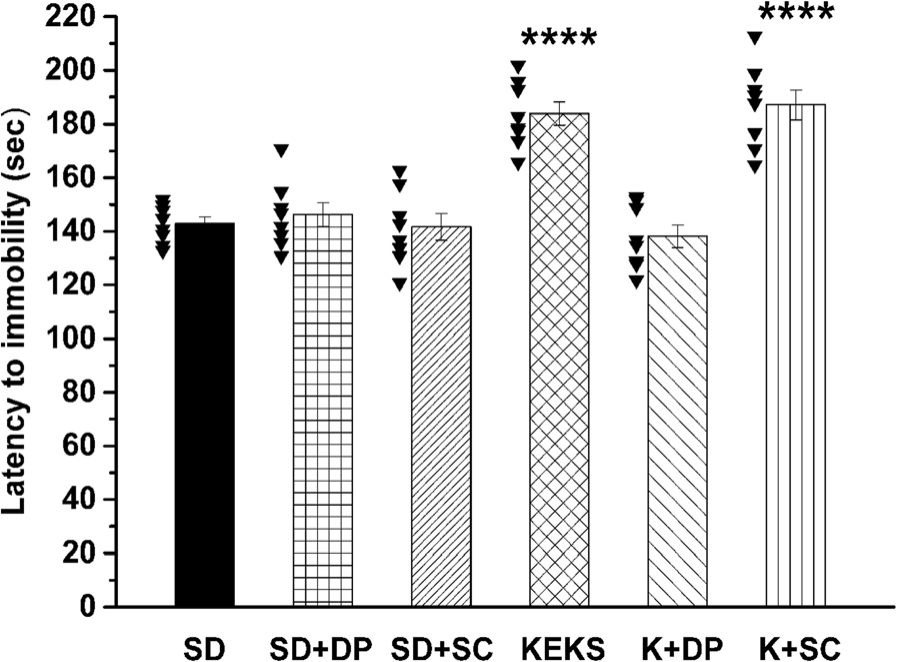
Influence of A1R antagonist DPCPX and A2AR antagonist SCH 58261 on KEKS-generated changes in isoflurane-evoked anesthesia (latency to immobility). Administration of DPCPX (i.p. 0.2 mg/kg; SD + DP; group 7) and SCH 58261 (i.p. 0.5 mg/kg; SD + SC; group 8) alone did not modulate the latency to immobility, compared to control (SD; the raw data was plotted as filled black triangles to the left of the columns). Combined administration of DPCPX (i.p. 0.2 mg/kg) with KEKS (K + DP; group 9) completely abolished the effect of KEKS on latency to immobility, whereas SCH 58261 (i.p. 0.5 mg/kg) was ineffective on the KEKS-induced influence (K + SC; group 10); Abbreviations: DP (DPCPX), 1,3-dipropyl-8-cyclopentylxanthine (a specific adenosine A1 receptor/A1R antagonist); KEKS, mix of KE and KS in a 1:1 ratio; SC (SCH 58261), 7-(2-phenylethyl)-5-amino-2-(2-furyl)-pyrazolo-[4,3-e]-1,2,4-triazolo[1,5-c] pyrimidine (a selective adenosine A2A receptor/A2AR antagonist); SD, standard diet/control; ****p < 0.0001

Administration of i.p. DPCPX (0.2 mg/kg; group 7) and SCH 58261 (0.5 mg/kg; group 8) alone (without KEKS administration) did not cause significant changes in the number of seconds required before isoflurane-induced anesthesia (latency to immobility), compared to control (SD; Fig. 3; Table 3) on the 7th day of gavage. It was demonstrated that i.p. 0.2 mg/kg DPCPX completely abolished the effect of KEKS on latency to immobility (group 9) (Fig. 3), whereas i.p. 0.5 mg/kg SCH 58261 (group 10) was ineffective on the KEKS-induced effect (Fig. 3; Table 3). After combined administration of KEKS with SCH 58261 on the 7th day of gavage, latency to immobility significantly increased, compared to control (SD; Fig. 3; Table 3) and both the rate of this increase and its significance level (*p* < 0.0001) was similar to results, which were recorded after gavage of KEKS alone (group 5) (Fig. 3; Table 3).

## Discussion

In this study we demonstrated that inhibition of A1Rs completely abolished the KEKS-evoked delay in isoflurane-induced anesthesia (immobility) in WAG/Rij rats. Moreover, we extended our previous results showing that not only gavage of KE and KS [23], but also KSMCT, KEKS and KEMCT are able to increase both the blood level of βHB (ketosis) and number of seconds required before induction of anesthesia (immobility).

Although isoflurane has been used in patients for nearly 50 years [19], its mechanism of action remains largely unknown. In spite of that both behavioral and physiological differences in functioning of sleep and general anesthetics-induced sleep-like state were demonstrated (e.g., general anesthesia is not able to appear spontaneously), it was suggested that several brain areas, such as cerebral cortex and the hypothalamic nucleus ventrolateral preoptic area may participate in both processes [42–44]. It was hypothesized, that anesthetics, such as isoflurane may induce anesthesia through common endogenous arousal neural circuitry/sleep pathways [44, 45].

Administration of exogenous ketone supplements by gavage and subsequent metabolism [17, 46, 47] increases levels of ketone bodies in the blood stream (nutritional ketosis) [1, 2, 4, 32]. Ketone bodies, such as βHB may enter into the brain through blood brain barrier and modulate different physiological and pathophysiological processes, such as sleep or seizures [7, 8, 12]. As ketosis (βHB) increases adenosine level [14] in the brain tissue and adenosine has a role in the sleep generation [28, 29], enhanced level of βHB generated by ketone supplements may modulate naturally occurring sleep. Indeed, exogenous ketone supplement-generated ketosis may evoke a decrease in total sleep time through ventrolateral preoptic area [20, 21, 44]. Moreover, it has been demonstrated that level and metabolism of both ketone bodies [7, 18, 48], as well as adenosine and expression of adenosine receptors [49] are regionally different in the brain, which strengthen the modulatory role of ketone bodies and adenosine in processes such as sleep and sleep-like states. Ketosis-evoked increase in extracellular adenosine levels may change neuronal activity in different brain areas [22, 49] implicated in sleep/sleep-like effects by its receptors. Increased level of adenosine was demonstrated during waking whereas adenosine concentration decreased during sleep in the brain [50]. Adenosine agonists induced sleep/electroencephalographic slow-wave activity, but adenosine receptor antagonists (e.g., a non-selective antagonist of adenosine receptors caffeine) reversed effects of adenosine on the sleep [51]. Moreover, adenosine accumulates under, for example, sleep deprivation and may have a role in the anesthetic action of isoflurane [28, 44]: theophylline (a non-selective antagonist of adenosine receptors) reversed the cerebral effects of isoflurane in dogs (e.g., EEG has been changed from a sleep pattern to an awake pattern) [30] and caffeine accelerated emergence from isoflurane-evoked anesthesia in humans [52]. Moreover, enhanced activity of A1Rs (e.g., by an A1R agonist N-sulfophenyl adenosine) may cause increase in anesthesia recovery time [53] and isoflurane may activate A1Rs [54]. It has been demonstrated that receptors of adenosine, such as inhibitory A1Rs and excitatory A2ARs are expressed brain areas implicated in the generation of sleep and sleep-like effects, such as ventrolateral/lateral preoptic area and basal forebrain [29]. Thus, adenosine may be a link between the anesthetic actions of isoflurane and sleep regulation as an endogenous sleep factor.

It was also demonstrated that inhibition or disinhibition by A1Rs (e.g., in wake-promoting neurons of basal forebrain or sleep-active neurons of ventrolateral preoptic area, respectively) may induce sleep [29, 55, 56]. Nevertheless, A1Rs may also promote wakefulness by inhibition of sleep-active neurons in lateral preoptic area [57] and in ventrolateral preoptic area [58]. Consequently, we can hypothesize that adenosinergic system may modulate the influence of exogenous ketone supplements, such as KEKS, on the onset of isoflurane-induced anesthesia (immobility) by inhibition of sleep active neurons (possibly by ketosis and, as a consequence, through increase in adenosine level as well as its A1Rs) [14, 23], which processes lead to delay in the anesthetic effects of isoflurane. Indeed, although the A1R antagonist DPCPX alone did not change the isoflurane-generated anesthetic effect (immobility), combined administration of DPCPX with KEKS completely abolished the KEKS-evoked increase in latency to immobility under isoflurane anesthesia (Fig. 3). Moreover, adenosine receptors may also modulate anesthesia recovery time [52, 53]. Thus, it is possible that exogenous ketone supplements not only delay the onset of isoflurane-induced anesthesia (immobility) [23] (Fig. 2a and 3), but also modulate the time required for recovery from anesthesia. However, further studies are needed to determine the exact effect and mechanism(s) of action of exogenous ketone supplements (ketosis) on isoflurane-generated anesthetic effects.

As it was demonstrated, not only A1Rs but also A2ARs are implicated in sleep generation, and A2ARs are considered more important in sleep regulation [29]: increased activity of A2ARs, for example, in ventrolateral/lateral preoptic area may induce sleep through sleep-active/promoting neurons [57, 59]. It has been demonstrated that A1Rs are abundantly expressed in the brain whereas A2AR expression is week in most of brain areas such as ventrolateral preoptic area [29, 49, 60]. Thus, as it was demonstrated, effects of adenosine on both sleep [29, 55–58] and processes of anesthesia may be brain region- and receptor-dependent. In addition, our knowledge relating to the exact role of adenosinergic system in modulation of both isoflurane-evoked anesthesia and connections between brain areas implicated in processes of anesthesia is far from complete. Consequently, it is possible that A1Rs are predominant whereas A2ARs are secondary (if any) in adenosine-evoked influences on anesthesia at least at this level of isoflurane-generated anesthesia (loss of consciousness without movement: immobility) in WAG/Rij rats. Indeed, our results suggest that A2ARs have no effect on isoflurane-generated anesthesia (immobility): neither isoflurane-induced anesthesia (latency to immobility) nor the effect of exogenous ketone supplement KEKS on isoflurane-induced anesthesia (latency to immobility) were modulated by the A2AR antagonist SCH 58261 (Fig. 3). Nevertheless, it cannot be excluded that this physiologically effective dose of A2AR antagonist SCH 58261 (0.5 mg/kg) may not have been adequate to investigate its influence on isoflurane-generated light phase of anesthesia (loss of consciousness without movement, immobility), but may modulate the later/deeper phase(s) of isoflurane-evoked anesthesia. However, more studies are needed to explain the exact role of adenosine and its receptors in isoflurane-induced anesthesia.

It has been demonstrated that gavage of exogenous ketone supplements, such as KSMCT for 7 days not only increases the number of seconds required before isoflurane-induced anesthetic induction (the time until immobility) (Fig. 2a) [23], but also generates decrease in both anxiety level on elevated plus maze [36] and absence epileptic activity [32] in WAG/Rij rats. These effects may be in correlation with enhanced level of βHB [23, 32, 36] (Fig. 2b and c). Moreover, it was showed that inhibition of A1Rs may abolish the anti-anesthetic (Fig. 3), antiepileptic [32] and anxiolytic [36] effects of exogenous ketone supplements, suggesting that adenosinergic system may modulate the ketone supplements (ketosis) induced influences in the CNS. Indeed, it was proposed that adenosinergic system (e.g., through A1Rs) has a role in the modulation of sleep and sleep-like effects [28–30], different types of epilepsies [61–63] and anxiety [64–66]. However, new studies are needed to reveal the likely (at least partly) common mechanism(s), as well as interactions of adenosine receptors and adenosine receptor-evoked changes (e.g., in ion channels, signal transduction, metabolic processes) in different brain areas involved in sleep/sleep-like effects, epilepsy and anxiety, by which ketone supplements could exert its above mentioned influences.

One limitation of our study is that we used the WAG/Rij rat strain exclusively to investigate the effect of ketone supplementation on isoflurane-induced anesthesia. In addition, during this study we narrowed our focus on the influence of ketone supplement-evoked effects to the adenosinergic system. Nevertheless, this WAG/Rij rat strain is extensively used for investigation of different drugs on CNS diseases [1, 67–71], and the present study further supports our previous experiments [23] on the role of the adenosinergic system. It has been suggested that the ketosis/βHB-evoked increase in adenosine levels [14] can modulate influence of ketone supplements not only on different CNS diseases [8, 32, 36], but also sleep and sleep-like effects [20, 21, 28–30] via adenosinergic system (likely through A1Rs). Consequently, we propose that the adenosinergic system may be one of main neurotransmitter systems by which ketone supplements can exert their influence on isoflurane-induced anesthesia. However, to get comprehensive view on influence of ketone supplements on isoflurane-evoked anesthesia more studies are needed on other animal strains and humans, on changes not only in adenosinergic, but also other neuromodulatory/neurotransmitter systems (such as cholinergic, dopaminergic, and GABAergic system), and on other phases of anesthesia/emergence from anesthesia [24, 53, 72] by administration of different/higher doses and types of ketone supplements. Further studies are also needed to reveal exact effects of different doses of drugs were used, such as DPCPX and SCH 58261, on isoflurane-generated anesthesia by administration of distinct methods (e.g., not only i.p., but also microinjections/microdialysis to specific brain areas, such as basal forebrain, as well as intravenous administration) [57, 58, 73].

## Conclusion

The present study strengthened the putative clinical and surgical relevance of ketone supplements-evoked influences on sleep and sleep-like effects suggested by our previous results: exogenous ketone supplements may increase the resistance to the isoflurane-induced anesthetic influence by delaying the onset of anesthesia (immobility). Thus, monitoring of blood ketone levels in humans undergoing isoflurane and (theoretically) other inhalational anesthesia may be important and helpful for the anesthesiologists. Moreover, inhibitory effect of DPCPX on KEKS-evoked delay in isoflurane-generated anesthesia (immobility) suggests that the adenosinergic system, likely via A_1_Rs, may modulate the exogenous ketone supplements-evoked anti-anesthetic influence. However, further studies are needed to reveal the exact mechanism(s) of action of exogenous ketone supplements (ketosis) on isoflurane-generated anesthesia not only in animals, but also in human subjects because ketone supplements used in normal and pathological conditions may modify the time needed for anesthesia.

## Data Availability

The data used and/or analyzed during the current study available from the corresponding author on reasonable request.

## Abbreviations

A_1_R: Adenosine A_1_ receptor
A2AR: Adenosine A2A receptor
CNS: Central nervous system
DMSO: Dimethyl sulfoxide
DPCPX: 1,3-dipropyl-8-cyclopentylxanthine
i.p.: intraperitoneal
K_ATP_ channels: ATP-sensitive potassium channels
KE (ketone ester): 1,3 butanediol-acetoacetate diester
KEKS: (mix of KE and KS)
KEMCT: (mix of KE and MCT oil)
KS: ketone salt
KSMCT: (mix of KS and MCT oil)
MCT: medium chain triglyceride
SCH 58261: 7-(2-phenylethyl)-5-amino-2-(2-furyl)-pyrazolo-[4,3-e]-1,2,4-triazolo[1,5-c]pyrimidine
SD: Standard rodent chow diet
WAG/Rij: Wistar Albino Glaxo/Rijswijk
βHB: beta-hydroxybutyrate
